# Molecular Characterization and Genetic Diversity of Isolated Foot-and-Mouth Disease Viruses Circulating in Cattle in The Mekong Delta Provinces, Vietnam

**DOI:** 10.1155/vmi/6680850

**Published:** 2025-04-07

**Authors:** Nguyen Phuc Khanh, Tran Duy Khang, Nguyen Thanh Lam, Chau Thi Huyen Trang, Le Trung Hoang

**Affiliations:** Faculty of Veterinary Medicine, College of Agriculture, Can Tho University, Can Tho, Vietnam

**Keywords:** foot-and-mouth disease virus, Mekong Delta Provinces, molecular characterization, Vietnam

## Abstract

Foot-and-mouth disease (FMD) is a highly contagious viral disease of cloven-footed livestock caused by foot-and-mouth disease virus (FMDV). FMD has significant impacts on farmers and national economies. The evolution and mutation of FMDV have contributed to the emergence of new strains of FMDV. Sequences of VP1 from 11 FMDV isolates in the Mekong Delta Provinces were obtained by Sanger sequencing technology. The phylogenetic analysis of VP1 sequence elucidated that 8 FMDV isolates including O-VN-CTU-VL01 (PP897837), O-VN-CTU-VL02 (PP897838), O-VN-CTU-TV01 (PP897840), O-VN-CTU-TV02 (PP897841), O-VN-CTU-TV03 (PP897842), O-VN-CTU-TV04 (PP897844), O-VN-CTU-BT04 (PP897847), and O-VN-CTU-BT05 (PP897847) were clustered into Group 1. On the other hand, 3 FMDV isolates including O-VN-CTU-BT01 (PP897844), O-VN-CTU-BT02 (PP897844), and O-VN-CTU-BT03 (PP897844) were clustered into Group 2. In addition, the nucleotide and deduced amino acid sequences of VP1 in Group 1 were closely related to lineage Mya-98, topotype SEA, and Type O (89%–93% nucleotide identity and 91%–99% amino acid identity). The similarity of FMDV isolates in Group 2 was closely related to lineage Pan Asia, topotype ME-SA, Type O (91.13%–94.53% and 96%–99.44% nucleotide and amino acid similarities, respectively). Analysis of amino acid sequences of VP1 illustrated several substitution mutations detected at amino acid positions 133–158 (the main antigenic site) in lineages Mya-98 (O/SEA) and Pan Asia, (O/ME-SA). Notably, a substitution mutation at position M144V was detected in FMDVs O-VN-CTU-VL1 (PP897837) and FMDV O-VN-CTU-TV1 (PP897840). No recombinant events were detected at VP1 sequences. In brief, genetic analysis of VP1 nucleotide and amino acid sequences of isolated FMDVs contributed to detecting the mutation which was able to cause the emergence of new strains as well as to elucidate the evolution of FMDVs circulating in the Mekong Delta Provinces.

## 1. Introduction

Foot-and-mouth disease (FMD) is a highly contagious disease of cloven-hoofed animals. The disease was initially described in the 16^th^ century and was the first animal pathogen identified as a virus [1]. FMD is classified in List A of OIE-listed diseases because it is capable of rapidly infecting and spreading within and between countries, resulting in great economic losses [2]. The causative agent of FMD is foot-and-mouth disease virus (FMDV), a member of the genus Aphthovirus in the family Picornaviridae, which is a nonenveloped, icosahedral virus 26–30 nm in diameter containing a single positive‐sense RNA genome approximately 8300 nucleotides in length [3]. The FMDV genome encodes a single polyprotein composed of four structural proteins (VP1–VP4) and ten nonstructural proteins (L, 2A-2C, 3A, 3B1–3B3, 3C, and 3D). Four structural proteins (VP1–4) contribute to the formation of the complete viral capsid. The structural proteins VP1, VP2, and VP3 are located on the surface of the virus, while VP4 is located on the inside of the virus. Among viral proteins, VP1 is closely related to viral attachment, protective immunity, and serotype specificity [4].

FMDV has a high degree of genetic and antigenic variation, which accounts for the existence of seven main FMDV serotypes, namely, O, A, C, Asia 1, Southern African Territories (SAT) 1, SAT 2, and SAT 3 [5, 6] and many subtypes [7]. FMDV Serotype O is classified into 11 topotypes, designated as Europe–South America (Euro–SA), Middle East–South Asia (ME–SA), Southeast Asia (SEA), Cathay (CHY), West Africa (WA), East Africa 1 (EA-1), East Africa 2 (EA-2), East Africa 3 (EA-3), East Africa 4 (EA-4), Indonesia-1 (ISA-1), and Indonesia-2 (ISA-2) [8, 9]. In addition, some serotypes have a restricted geographical distribution, e.g., Asia-1, whereas others, notably Serotype O, occur in many different regions. There is no cross-protection between serotypes and sometimes protection conferred by vaccines even of the same serotype can be limited [10].

In Vietnam, the FMDV Serotype O and its outbreak were first reported in academic research between 1996 and 2001 [11]. In addition, the FMDV Serotypes Asia 1 and A were subsequently identified in 2005 and 2009, respectively [12, 13]. In 2008 and 2009, FMDV Serotypes O and A were reported to be the prevalent serotype and caused almost FMD outbreaks. Then, the first detection of FMDV O/Ind-2001d in 2017 was reported in [14]. Moreover, cattle are commonly vaccinated against FMDV Serotype O twice annually with varying vaccine formulations, based upon current knowledge of circulating strains. However, sporadic FMD outbreaks have been reported in the Mekong Delta Provinces. Prominent factors of the disease are poor cross-protection between serotypes, rapid spread, and the diversity of serotypes. Currently, limited information is available regarding the genetic characteristics and geographical distribution of the FMDV causing sporadic outbreaks in the Mekong Delta Provinces. In this study, the VP1 nucleotide and amino acid sequences of 11 FMDV isolates collected from endemic outbreaks in some provinces in the Mekong Delta Provinces (Southern Vietnam) during 2022–2023 were analyzed to investigate their phylogeny and genetic relationship with other published FMDVs.

## 2. Materials and Methods

### 2.1. Ethical Approval

All experimental protocols were approved by the Institutional Animal Care and Use Committee of Can Tho University, Vietnam.

### 2.2. Sample Collection

**Figure 1:**
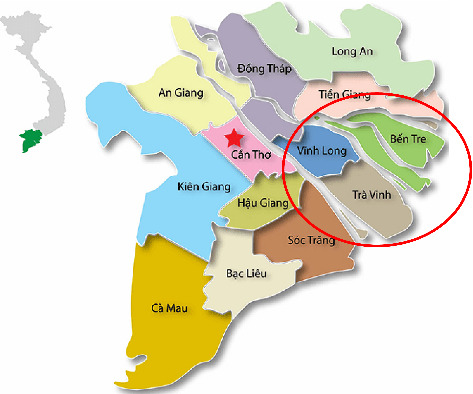
The Mekong Delta map shows the areas where samples were collected. Samples were collected from 3 provinces consisting of Ben Tre (10°10′N, 106°30′E), Tra Vinh (9°50′N, 106°15′E), and Vinh Long(10°10′N, 106°0′E).

The samples were collected and processed following the World Organization for Animal Health (OIE) standard criteria [15, 16]. A total of 40 samples (damaged skin, vesicles, and/or probang) were collected from cattle exhibiting symptoms of FMD (Table 1) in FMD outbreaks located in the Mekong Delta Provinces consisting of Ben Tre, Tra Vinh, and Vinh Long Provinces. The location of those provinces is shown in Figure 1. The collected samples were stored in ice bins on the way to the laboratory and stored in the freezer (−70°C).

### 2.3. Primer Design

Primer design was performed as described previously in [17]. Two sets of gene-specific primers shown in Table 2 are designed using NCBI Primer3 (available on the website: https://www.ncbi.nlm.nih.gov/tools/primer-blast/) and BLAST tools (available on the website: https://blast.ncbi.nlm.nih.gov). Then, the specificity of the designed primers was evaluated based on the aligning primers with reference FMDV strain O/YS/CHA/05 (HM008917) using MEGA 6.06 software.

### 2.4. FMD Screening Using Real-Time Reverse Transcription-Polymerase Chain Reaction (qRT-PCR)

#### 2.4.1. RNA Extraction

Total RNA was extracted from collected samples using an RNA extraction kit (NEXprep™, Korea) as described by the manufacturer's instructions.

#### 2.4.2. cDNA Synthesis

First-strand cDNA was synthesized using a Sensi FAST™ cDNA synthesis kit (Bioline, UK). In brief, a 20-μL total reaction volume including 10 μL of RNase-free water, 5 μL of extracted RNA sample, 1 μL of reverse transcriptase primer, and 4 μL of 5x TransAmp Buffer was prepared in 1.5-mL Eppendorf tube and kept on ice. Then, the reaction was incubated at 25°C for 10 min followed by heating at 45°C for 10 min and 85°C for 5 min and chilled on ice for at least 1 min. The synthesis cDNA was immediately used for real-time PCR or PCR amplification.

#### 2.4.3. Real-Time PCR

For the detection of FMDV, primers directed toward the conserved UTR are used as previously described in [18] (Table 2). 2x SensiFAST™ SYBR (Bioline, UK) was used for UTR amplification, following the manufacturer's instructions. The PCR master mix was prepared in a total volume of 15 μL. It consisted of 3.4 μL of dH2O, 10 μL of 2x SensiFAST™ SYBR, and 0.8 μL of each primer (10 pmol/μL). Template cDNA was added at 5 μL for a complete volume of 20 μL. The mixture was placed in FQD-96A (Bioer, USA) with the following cycling conditions: initial denaturation (95°C for 120 s) and 40 cycles of amplification (95°C for 5 s, 60°C for 10 s, and 72°C for 15 s) and ending with a melting curve from 60°C to 90°C.

### 2.5. RT-PCR

RT-PCR was carried out to amplify VP1 of FMDVs. The forward and reverse primers used in this assay are shown in Table 2.

The PCR was performed by using a MyTaq™ DNA Polymerase kit (Bioline, UK) according to the manufacturer's instructions. In brief, 20 μL of master mix including 6.5 μL of nuclease-free water, 12.5 μL of MyTaq Mix 2X, and 1 μL of 20 μM forward and reverse primers were combined with 5 μL of cDNA to reach a total of 25 μL reaction. Subsequently, PCRs were performed following the protocol: 1 cycle of initial denaturation at 95°C for 15 s, a sequence of 35 cycles followed by denaturation at 95°C for 15 s, annealing at 60°C for 15 s, extension at 72°C for 30 s, and 1 cycle of final extension 72°C for 3 min. The PCR products were detected on 1%, 5% (w/v) agarose gel electrophoresis, and the gel picture was captured by using a UV light transilluminator (UVDI, USA).

### 2.6. Sequencing and Phylogenetic Analysis

Sequencing was performed in both directions with virus-specific primers. Sequences were analyzed using the Genetyx Version 12. This program was used to read the sequencing electropherograms to exclude nucleotide ambiguity. Sequences of FMDV trains were aligned with the ClustalW multiple alignment method using BioEdit 7.2.0 [19, 20]. Phylogenetic analyses were conducted using MEGA 6.06 [21], and phylogenetic trees were constructed based on strains in various geographic origins and topotypes (strains and accession numbers were retrieved from GenBank) by using the maximum likelihood method based on the Tamura Parameter 3 model with 1000 bootstrap replicates.

### 2.7. Recombination Analysis

Recombination analysis was performed as described previously in [17]. Briefly, eleven FMDV strains and reference FMDVs were performed for recombination analysis by using Simplot v3.5 and recombinant detection program v4.70 (RDP4). Both programs were applied to identify putative recombination breakpoints and parental isolates of recombinants. The Simplot program is performed based on a sliding window method. Nucleotide identity was calculated using the Kimura 2-parameter method with a transition–transversion ratio of 2, and the window width and step size, respectively, were 200 bp and 20 bp. Bootscan analysis was also carried out using a signal of 70% of the observed permuted tree. In the RDP4 program, various potential recombination methods including RDP, Geneconv, Bootscan, MaxChi, Chimaera, Siscan, and Topal DSS were used to identify the recombination events and parental isolates of recombinants, with significance set at *p* values < 0.05.

## 3. Results

### 3.1. FMDV Screening Using Real-Time RT-PCR

Out of 40 collected samples, 11 samples were positive with FMDV accounting for 27.50%. Positive samples had Ct values ranging from 15.45 to 25.59 (Table 3). During the sample collection, the FMDV-positive cattle observed salivation, pyrexia, and vesicle formations in the buccal and nasal mucous membranes and/or between the claws, and lameness.

### 3.2. Phylogenetic Analysis and Pairwise Sequence Comparison Analysis (PSCA) of VP1

**Figure 2:**
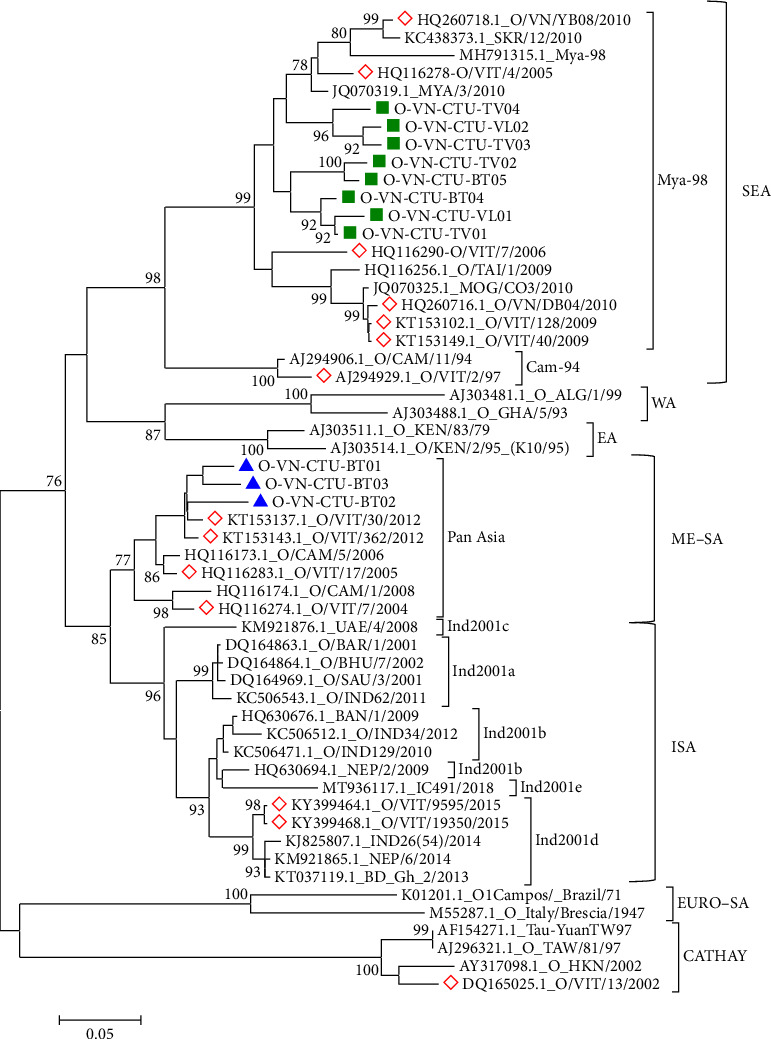
Phylogenetic relationships of the FMDV isolates and published FMDVs based on VP1 nucleotide sequences determined using MEGA 6.06 with the ClustalW method. Green squares and blue triangles indicate isolated FMDV Groups 1 and 2, respectively; red diamonds indicate FMDV strains circulating in Vietnam.

In the present study, the phylogenetic relationship between the sequences of 11 FMDV isolates and 44 published FMDVs was assessed based on the VP1 sequence at Positions 1–633. The results showed that isolates O-VN-CTU-VL01 (PP897837), O-VN-CTU-VL02 (PP897838), O-VN-CTU-TV01 (PP897840), O-VN-CTU-TV02 (PP897841), O-VN-CTU-TV03 (PP897842), O-VN-CTU-TV04 (PP897844), O-VN-CTU-BT04 (PP897847), and O-VN-CTU-BT05 (PP897847) clustered into Group 1 (90.33%–97.96% nucleotide identity and 94.12%–99.44% amino acid identity) while isolates O-VN-CTU-BT01 (PP897844), O-VN-CTU-BT02 (PP897844), and O-VN-CTU-BT03 (PP897844) clustered into Group 2 (92.65%–95.82% nucleotide identity and 96.60%–98.88% amino acid identity). According to the phylogenetic tree (Figure 2), isolates in Group 1 had a close relationship to lineage Mya-98, topotype SEA, and Type O (O/SEA). In addition, isolates in Group 2 shared a close relationship to lineage Pan Asia, topotype ME-SA, and Type O (O/ME-SA).

PSCA of VP1 nucleotide and amino acid sequences showed that Group 1 shared high identity with those strains in lineage Mya-98 (O/SEA) including O/VIT/4/2005 (HQ116278, Vietnam), MYA/3/2010 (JQ070319.1, Myanmar), and SKR/12/2010 (KC438373.1, South Korea) with approximately 89%–93% nucleotide identity and 91%–99% amino acid identity. Those reference strains were reported in different parts of Northern Vietnam and Asian countries. Besides, those strains in Group 1 shared 87%–90% nucleotide identity and 92%–97% amino acid identity with MOG/CO3/2010 (JQ070325.1, Mongolia) and O/TAI/1/2009 (HQ116256.1, Thailand). Meanwhile, Group 2, respectively, shared 91.13%–94.53% and 96%–99.44% nucleotide and amino acid similarities with those strains in lineage Pan Asia (O/ME-SA) consisting of O/VIT/7/2004 (HQ116274.1, Vietnam) and O/CAM/5/2006 (HQ116173.1, Cambodia). In addition, phylogenetic and PSCA of VP1 sequences showed that all FMDV isolates distinctly separated from the strains in topotypes CHY, WA, EA, EURO–SA, and ISA with approximately 73%–90% nucleotide similarity and 83%–97% amino acid similarity (Table 4).

Those results illustrate the circulation of FMDVs lineage Mya-98 (O/SEA) and lineage Pan Asia (O/ME-SA) in the Mekong Delta Provinces, Vietnam.

**Figure 3:**
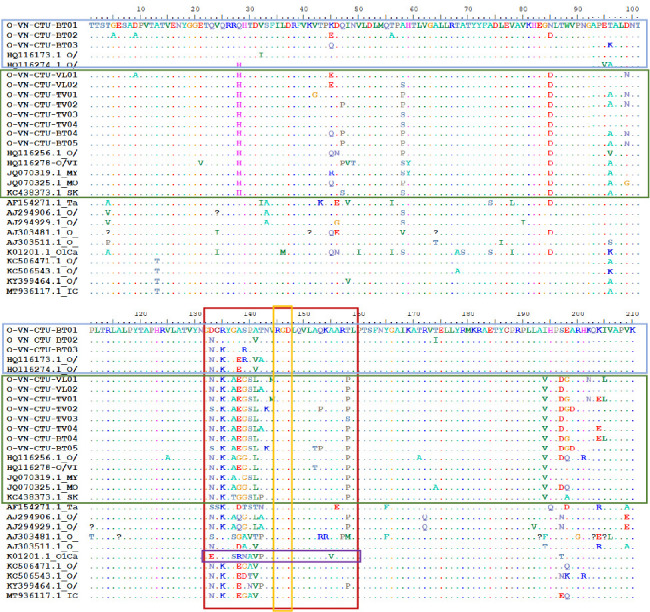
Amino acid sequences of antigenic regions in VP1 of FMDVs. Amino acid sequence alignments of VP1 from the 11 isolated strains and 17 representative reference strains for different lineages of Type O. Dots indicate the amino acid position; letters indicate amino acid changes. The blue and green boxes indicate the amino acid sequences of lineage Pan Asia and Mya-98, respectively. The red and orange boxes indicate the amino acid positions of neutralizing epitopes and cell attachment sites of FMDVs, respectively. The purple box indicates the positions of neutralizing epitopes in vaccine strain O1/Campos/Brazil (K01201.1).

Figure 3 shows that a total of 15/210 (7.14%) and 30/210 (14.29%) amino acid substitutions were recorded between the predicted VP1 protein of the isolated sequences and the reference sequences within the Pan Asia and Mya-98 lineages, respectively.

Comparison of amino acid sequences of the main antigenic site in the VP1 protein (amino acid positions 133–158) showed that there were some substitutions occurred at Positions 133, 135, 138, 139, 141, and 142 between isolated and FMDV reference strains in lineage Pan Asia and at Positions 137, 138, 140, 143, 144, 152, and 153 in lineage Mya-98. Compared to reference strains in other countries, a substitution mutation was detected at Position 139 in the isolated FMDVs. Remarkably, there was a substitution mutation at Position M144V in FMDVs O-VN-CTU-VL1 (PP897837) and FMDV O-VN-CTU-TV1 (PP897840). The result also showed that amino acid Positions 145–147 (RGD) of isolated and reference FMDV strains were completely conserved.

Furthermore, the amino acid differences of the isolated FMDVs with FMDV vaccine strain O1/Campos/Brazil (K01201.1) were compared in this study. Compared with the vaccine strain, the substitution at Positions 133, 137, 138, 139, 140, 141, 142, and 155 were detected in the isolated strains in lineage Pan Asia and at Positions 135, 137–144, 152, and 153 in lineage Mya-98.

### 3.3. Recombination Analysis

The negative recombinant events in VP1 sequences of isolated FMDVs were characterized by using a standard similarity plot (Simplot) and RDP.

## 4. Discussion

Among infectious diseases in cattle, FMD is one of the most important diseases of livestock and is endemic in several countries broadly. The FMD also causes economic losses to the livestock population and reduces food security and economic development. FMD has been prevalent in Vietnam since 1898 with occurrences of sporadic outbreaks from time to time. Several studies on FMD have been published in Northern Vietnam; however, no studies have determined the evolution of FMDVs through a complete analysis in the Mekong Delta Provinces. The interpretation of the phylogenetic relationships among the FMDV strains contributes to proper control measures against the disease. Therefore, the present study considered it necessary to fill the existing knowledge gap of characterization, evolution, and origins of FMDV strains. The study results would be critical for developing and deploying effective vaccines in the future.

The VP1 sequences are usually used to identify the molecular characterization of FMDV [22]. Besides, the VP1 capsid protein plays an important role in the investigating relationship between different FMDV strains because of its significance for protective immunity, serotype specificity, and attachment and entry of FMDV. In the present study, the VP1 nucleotide and amino acid sequences of the FMDV Serotype O from isolated strains in the Mekong Delta Provinces in Vietnam and published reference strains in the north and northern central regions of Vietnam and other Asian countries (China, Laos, Cambodia, Myanmar, Bangladesh, Mongolia, South Korea, and Thailand) (Table 1) were used for the identification of the phylogeny and genetic relationships between isolated FMDVs and reference strains.

In this study, phylogenetic analysis and PSCA showed that there was the circulation of FMDV lineage Mya-98 (O/SEA) (O-VN-CTU-VL01 [PP897837], O-VN-CTU-VL02 [PP897838], O-VN-CTU-TV01 [PP897840], O-VN-CTU-TV02 [PP897841], O-VN-CTU-TV03 [PP897842], O-VN-CTU-TV04 [PP897844], O-VN-CTU-BT04 [PP897847], and O-VN-CTU-BT05 [PP897847]); and lineage Pan Asia (O/ME-SA) (O-VN-CTU-BT01 [PP897844], O-VN-CTU-BT02 [PP897844], and O-VN-CTU-BT03 [PP897844]) in the Mekong Delta Provinces. This result is consistent with a previously published study in [23]; Pan Asia (O/ME-SA) and Mya-98 (O/SEA) were different lineages of Serotype O which have been circulating in Vietnam recently.

Topotype SEA was the most frequently detected in Type O and had two lineages, named Mya-98 and Cam-94 [24]. The isolated FMDV strain, lineage Mya-98 (O/SEA), shared a close relationship with those strains from the north and northern central regions of Vietnam and Asia countries consisting of China, South Korea, Myanmar, Thailand, and Mongolia. These findings are partially consistent with previous studies conducted by the OIE, which assessed the livestock movement in Vietnam and neighboring countries [25]. In addition, previous reports showed that Mya-98 (O/SEA) was normally restricted to all six mainland countries of the SEA region and has caused outbreaks in Myanmar (1998), Laos (1998), Cambodia (1998), Thailand (1999), Malaysia (2001), and Vietnam (2002) [22]. Besides, Mya-98 (O/SEA) has caused outbreaks in eastern and Central Asia, specifically Hong Kong (2010), South Korea (2010–2011), North Korea (2010), Japan (2010), Mongolia (2010), Russia (2010), and Taiwan (2012) [26–28].

Lineage Pan Asia (O/ME-SA) was first described in the 1980s in the Indian subcontinent and subsequently disseminated broadly throughout southern Asia, the Middle East, and East and Southeast Asia, and some incursions in South Africa and European countries [8]. Moreover, the Pan Asia lineage had previously been introduced into SEA specifically in Malaysia in 2003–2005 and became widespread along with the local Mya98 (O/SEA). Besides, Pan Asia was found during this period in Cambodia, Vietnam, Lao, China, and Thailand [29].

Our findings indicate that Mya-98 (O/SEA) and Pan Asia (O/ME-SA) are the predominant lineages or topotypes of FMDV Serotype O circulating in the Mekong Delta Provinces (southern Vietnam).

The analysis of amino acid sequences of VP1 indicates several substitution mutations detected in isolated FMDVs, especially, mutations at the antigenic site (amino acid position 133–158). According to [30], the peptide 136–151 plays an important role in neutralizing FMDV, and particularly amino acid 139 is a decisive factor in the neutralizing properties of the main antigen region in VP1. In this study, a substitution mutation at Position M144V was recorded in FMDVs O-VN-CTU-VL1 (PP897837) and FMDV O-VN-CTU-TV1 (PP897840). A previous study of [31] revealed that the amino acids 140–160 are the first neutralizing antigen site in VP1 of FMDV, in which the amino acids 144, 148, and 154 play a decisive role in the neutralizing properties of this antigenic site of FMDV. Furthermore, a change in any of the three amino acids 144, 154, or 208 can lead to a decrease in the ability to neutralize the VP1 antigen of FMDV [32]. Therefore, further study in FMDVs O-VN-CTU-VL1 (PP897837) and FMDV O-VN-CTU-TV1 (PP897840) should be performed. In the present study, 3 amino acid RGD at Positions 145–147 were detected in all FMDVs. According to [33], the amino acid sequence involved in the attachment of FMDV Serotype O to cells is located in regions 141–160 and 200–213, in which amino acids 145–147, 206, and 207 play a decisive role.

In Vietnam, cattle are compulsorily vaccinated against FMDV Serotype O twice annually with varying vaccine formulations, based on current knowledge of circulating strains. However, sporadic FMD outbreaks have been reported in southern Vietnam. This suggests there may be a vaccination failure. Possible causes of vaccination failure include the presence of immunosuppressive disorders, improper storage and transport of the vaccine, and immune evasion due to the presence of different vaccine variants as previously reviewed [34]. Besides, changes in the antigenic properties should be considered. According to [35–37], antigenic properties of FMDV strains are associated with specific amino acid motifs in the VP1 protein: 43–45, 83, 96, 141–160 (G-H loop), 169–173, and 200–211 (C-terminus). In this study, substitutions at Positions 133, 137–142, and 155 were detected in the isolated strains in lineage Pan Asia (O/ME-SA) and at Positions 135, 137–144, 152, and 153 in lineage Mya-98 (O/SEA). Hence, the possibility that the current vaccine strains may offer reduced protection against the recent circulating strains. Cross-neutralization studies should be assessed in further studies to evaluate the effectiveness of current vaccines in the prevention of FMD in southern Vietnam.

## 5. Conclusions

Molecular characterization of VP1 sequences of circulating FMDVs in the Mekong Delta Provinces, Vietnam, has provided valuable information on the circulation and evolution of FMDVs. Genetic analysis of nucleotide and amino acid sequences of VP1 illustrated substitution mutation at the antigenic region contributing to the emergence of new FMDVs in lineage Mya-98 (O/SEA) and Pan Asia (O/ME-SA) circulating in the Mekong Delta Provinces, Vietnam. Moreover, further studies about the pathogenicity of FMDVs O-VN-CTU-VL1 (PP897837) and FMDV O-VN-CTU-TV1 (PP897840) should be performed for insight knowledge about these isolates contributing to vaccine development for controlling the FMD.

## Figures and Tables

**Table 1:** GenBank accession numbers and sources of isolated and reference FMDV strains.

No.	Accession no.	Strain	Lineage	Topotype	Country	Year	Host
1	PP897837	O-VN-CTU-VL01			Vietnam: Vinh Long	2023	Cattle
2	PP897838	O-VN-CTU-VL02			Vietnam: Vinh Long	2023	Cattle
3	PP897840	O-VN-CTU-TV01			Vietnam: Tra Vinh	2023	Cattle
4	PP897841	O-VN-CTU-TV02			Vietnam: Tra Vinh	2023	Cattle
5	PP897842	O-VN-CTU-TV03			Vietnam: Tra Vinh	2023	Cattle
6	PP897843	O-VN-CTU-TV04			Vietnam: Tra Vinh	2023	Cattle
7	PP897844	O-VN-CTU-BT01			Vietnam: Ben Tre	2023	Cattle
8	PP897845	O-VN-CTU-BT02			Vietnam: Ben Tre	2023	Cattle
9	PP897846	O-VN-CTU-BT03			Vietnam: Ben Tre	2023	Cattle
10	PP897847	O-VN-CTU-BT04			Vietnam: Ben Tre	2022	Cattle
11	PP897848	O-VN-CTU-BT05			Vietnam: Ben Tre	2022	Cattle
12	HQ260718.1	O/VN/YB08/2010	Mya-98	SEA	Vietnam: Yen Bai	2010	Cattle
13	KC438373.1	SKR/12/2010	Mya-98	SEA	South Korea	2010	Cattle
14	MH791315.1	Mya-98	Mya-98	SEA	China	2018	Cattle
15	HQ116278	O/VIT/4/2005	Mya-98	SEA	Vietnam	2005	Cattle
16	JQ070319.1	MYA/3/2010	Mya-98	SEA	Myanmar	2010	Cattle
17	HQ116290	O/VIT/7/2006	Mya-98	SEA	Vietnam	2006	Cattle
18	HQ116256.1	O/TAI/1/2009	Mya-98	SEA	Thailand	2009	Cattle
19	KT153102.1	O/VIT/128/2009	Mya-98	SEA	Vietnam: Ha Giang	2009	Cattle
20	HQ260716.1	O/VN/DB04/2010	Mya-98	SEA	Vietnam: Dien Bien	2010	Cattle
21	JQ070325.1	MOG/CO3/2010	Mya-98	SEA	Mongolia	2010	Cattle
22	KT153149.1	O/VIT/40/2009	Mya-98	SEA	Vietnam: Ha Tinh	2009	Cattle
23	AJ294906.1	O/CAM/11/94	Cam-94	SEA	Cambodia	1998	Cattle
24	AJ294929.1	O/VIT/2/97	Cam-94	SEA	Vietnam	1997	Cattle
25	AJ303481.1	O ALG/1/99		WA	Algeria	1999	—
26	AJ303488.1	O GHA/5/93		WA	Ghana	1993	—
27	AJ303511.1	KEN/83/79		EA	Kenya	1979	—
28	AJ303514.1	O/KEN/2/95		EA	Kenya	1995	—
29	KT153137.1	O/VIT/30/2012	Pan Asia	ME-SA	Vietnam: Son La	2012	Cattle
30	KT153143.1	O/VIT/362/2012	Pan Asia	ME-SA	Vietnam: Long An	2012	Cattle
31	HQ116173.1	O/CAM/5/2006	Pan Asia	ME-SA	Cambodia	2006	Cattle
32	HQ116283.1	O/VIT/17/2005	Pan Asia	ME-SA	Vietnam	2005	Cattle
33	HQ116174.1	O/CAM/1/2008	Pan Asia	ME-SA	Cambodia	2008	Cattle
34	HQ116274.1	O/VIT/7/2004	Pan Asia	ME-SA	Vietnam	2004	Cattle
35	KM921876.1	UAE/4/2008	Ind2001c	ISA	United Arab Emirates	2008	Gazelle
36	DQ164864.1	O/BHU/7/2002	Ind2001a	ISA	Bhutan	2002	
37	DQ164969.1	O/SAU/3/2001	Ind2001a	ISA	Saudi Arabia	2001	Cattle
38	DQ164863.1	O/BAR/1/2001	Ind2001a	ISA	Bahrain	2001	Cattle
39	KC506543.1	O/IND62/2011	Ind2001a	ISA	India: Maharashtra	2001	Cattle
40	HQ630676.1	BAN/1/2009	Ind2001b	ISA	Bangladesh	2009	Cattle
41	KC506512.1	O/IND34/2012	Ind2001b	ISA	India: Karnataka	2011	Cattle
42	HQ630694.1	NEP/2/2009	Ind2001b	ISA	Nepal: Kathmandu	2009	Cattle
43	KC506471.1	O/IND129/2010	Ind2001b	ISA	India: Assam	2010	Cattle
44	MT936117.1	IC491/2018-	Ind2001e	ISA	India: Karnataka	2018	Cattle
45	KY399464.1	O/VIT/9595/2015	Ind2001d	ISA	Vietnam: Dak Nong	2015	Cattle
46	KY399468.1	O/VIT/19350/2015	Ind2001d	ISA	Vietnam: Ninh Thuan	2015	Cattle
47	KJ825807.1	IND26(54)/2014	Ind2001d	ISA	India: Assam	2014	Cattle
48	KT037119.1	BD Gh 2/2013	Ind2001d	ISA	Bangladesh	2013	Cattle
49	KM921865.1	NEP/6/2014	Ind2001d	ISA	Nepal: Ilam	2014	Cattle
50	K01201.1	O/Campos/Brazil/71	—	EURO-SA	Brazil	1971	—
51	M55287.1	O/Italy/Brescia/1947	—	EURO-SA	Italy	1947	—
52	AF154271.1	Tau-YuanTW97	—	CATHAY	Taiwan	1997	Pig
53	AJ296321.1	O/TAW/81/97	—	CATHAY	Taiwan	1997	Pig
54	AY317098.1	O/HKN/2002	—	CATHAY	China	2002	Pig
55	DQ165025.1	O/VIT/13/2002	—	CATHAY	Vietnam	2002	Pig

**Table 2:** Primers employed for amplification of UTR and VP1 sequences.

No.	Primers	Sequence 5′-3′	Location	Product size (bp)
*Real-time RT-PCR, UTR* [18]				
1	FP	GTA AAC CAC TGG TTG GCT GAC T		56
	RP	TAC TCG CCG TGG CCT CG		

*RT-PCR, VP1*				
2	FP	GAC TTY GAG YTR CGB YTR CC	3374–3394	744
	RP	GGG TTG GAC TCV ACG TCY CC	4118–4088	

**Table 3:** History of FMDV-positive cases and the accession numbers of the VP1 sequences of FMDV isolates.

No.	Accession no.	Isolates	Origin	Cycle threshold	Clinical signs	Type of sample
1	PP897837	O-VN-CTU-VL01	Vinh Long	23.60	Pyrexia (> 41°C), blisters in the buccal and nasal mucous membranes	Damaged skin and vesicles
2	PP897838	O-VN-CTU-VL02	Vinh Long	21.69	Pyrexia (> 41°C), blisters in the buccal and nasal mucous membranes	Damaged skin and vesicles
3	PP897840	O-VN-CTU-TV01	Tra Vinh	24.74	Pyrexia (> 41°C), drooling	Probang
4	PP897841	O-VN-CTU-TV02	Tra Vinh	15.45	Blisters in the buccal and nasal mucous membranes and between the claws, lameness	Damaged skin and vesicles
5	PP897842	O-VN-CTU-TV03	Tra Vinh	22.64	Pyrexia (> 41°C), drooling	Probang
6	PP897843	O-VN-CTU-TV04	Tra Vinh	23.38	Blisters between the claws, lameness	Damaged skin and vesicles
7	PP897844	O-VN-CTU-BT01	Ben Tre	23.01	Pyrexia (> 41°C), blisters in the buccal and nasal mucous membranes	Damaged skin and vesicles
8	PP897845	O-VN-CTU-BT02	Ben Tre	24.90	Pyrexia (> 41°C), blisters between the claws, lameness	Damaged skin and vesicles
9	PP897846	O-VN-CTU-BT03	Ben Tre	25.12	Pyrexia (> 41°C), blisters between the claws, lameness	Damaged skin and vesicles
10	PP897847	O-VN-CTU-BT04	Ben Tre	25.59	Pyrexia (> 41°C), drooling	Probang
11	PP897848	O-VN-CTU-BT05	Ben Tre	21.20	Pyrexia (> 41°C), drooling	Probang

**Table 4:** Nucleotide and amino acid similarities of VP1 sequence between the isolated and reference FMDV strains.

	Amino acid similarity (%)																											
	1	2	3	4	5	6	7	8	9	10	11	12	13	14	15	16	17	18	19	20	21	22	23	24	25	26	27	28
1		97.14	98.28	95.31	96.56	96.55	92.13	91.54	93.37	97.12	94.70	93.43	93.40	94.06	92.79	95.90	95.84	95.88	86.10	92.92	91.00	86.07	90.83	87.56	92.74	92.23	92.90	92.84
2	91.69		95.91	95.32	99.44	99.44	94.05	93.47	95.26	97.13	94.72	95.31	95.28	95.98	95.90	98.86	97.13	98.26	87.54	95.97	94.15	88.73	92.79	90.88	94.11	93.58	94.76	94.19
3	97.96	93.03		94.65	95.32	96.52	90.07	89.47	91.36	97.69	94.04	91.44	91.39	93.39	91.43	94.63	95.18	94.60	84.26	91.57	89.60	85.68	89.21	86.05	90.71	90.20	90.90	90.83
4	92.72	91.13	92.89		94.72	94.71	89.92	88.83	90.72	97.11	99.43	90.81	90.75	92.77	92.11	94.02	94.57	94.67	84.47	90.95	88.96	85.55	88.08	86.05	90.07	89.56	90.27	90.20
5	90.73	97.78	92.09	90.73		98.87	94.10	93.53	95.31	96.54	94.12	95.35	95.33	95.39	95.30	98.28	96.54	97.68	87.65	95.39	93.55	88.78	92.86	90.96	94.17	93.64	94.17	94.25
6	90.93	95.10	92.28	91.12	95.29		93.42	92.84	94.64	97.71	94.10	94.69	94.66	95.38	95.29	98.28	96.53	97.67	87.31	95.37	93.53	89.45	92.64	90.22	93.49	92.96	94.15	93.58
7	80.58	83.56	80.87	80.15	82.42	81.54		96.60	98.88	91.45	89.26	98.32	97.17	91.52	92.24	94.13	92.73	93.45	89.12	95.29	93.41	90.67	96.60	92.48	96.60	95.40	95.81	95.94
8	83.59	82.70	82.32	80.92	82.48	81.59	93.78		96.60	90.86	88.17	97.17	96.00	90.94	91.66	93.56	92.57	92.88	88.24	93.51	91.60	89.38	95.43	92.10	95.42	94.20	94.58	94.74
9	82.91	82.65	81.58	81.10	81.96	81.54	95.82	92.65		92.71	90.07	99.44	98.31	92.77	93.47	95.33	93.98	94.67	89.90	96.49	94.64	90.17	96.60	91.72	97.75	96.57	97.01	97.12
10	96.20	93.40	97.96	92.86	92.46	92.07	81.55	82.98	82.24		96.52	92.77	92.73	94.67	94.04	95.89	96.44	96.51	85.79	92.89	90.97	87.23	90.62	87.51	92.07	91.56	92.24	92.18
11	93.10	91.13	93.27	97.60	90.33	90.93	80.64	81.40	80.87	93.62		90.17	90.11	92.15	92.74	93.40	93.95	94.06	83.78	90.32	88.31	84.83	87.41	85.38	89.42	88.92	89.63	89.56
12	83.55	85.73	83.82	82.45	84.20	83.79	94.53	92.85	94.36	85.12	83.37		98.88	92.84	93.53	95.37	94.03	94.72	89.81	96.52	94.69	90.07	97.17	92.33	98.31	97.15	97.61	97.70
13	83.75	85.06	83.80	83.57	84.40	84.66	92.09	91.13	93.05	84.66	84.25	93.60		92.80	93.50	95.35	94.00	94.70	88.48	95.31	93.44	88.71	96.00	91.06	97.16	95.97	96.39	96.51
14	89.11	87.57	88.86	88.90	87.58	87.57	80.55	81.09	81.50	90.48	89.32	82.15	82.82		92.83	94.71	96.54	94.63	84.22	92.96	91.63	85.55	90.22	88.21	92.22	92.28	91.35	92.32
15	90.56	90.31	91.72	90.93	89.90	91.11	81.36	81.41	81.59	92.07	91.32	83.15	82.91	88.88		97.13	94.03	95.96	85.52	93.54	91.64	85.97	90.96	87.69	91.67	90.36	93.30	90.88
16	91.52	92.27	92.86	92.11	91.88	93.04	82.65	82.70	82.42	92.85	92.11	84.46	83.32	89.88	96.55		95.89	98.29	87.70	95.38	93.54	88.88	92.89	90.38	94.19	92.97	94.84	93.47
17	88.93	87.62	88.69	88.31	87.62	87.40	82.47	82.53	83.16	90.11	88.73	83.57	83.32	96.00	88.08	89.29		95.83	85.11	94.13	92.24	86.45	91.44	88.16	93.41	92.22	92.57	93.50
18	90.16	89.91	91.33	91.91	89.29	89.70	80.42	81.42	81.13	91.68	90.73	83.16	82.23	87.83	93.04	93.61	87.45		87.52	94.73	92.86	88.06	92.19	89.64	94.11	92.85	94.18	93.37
19	73.75	75.70	74.26	74.00	76.20	75.94	78.99	78.08	79.46	75.25	73.75	80.12	79.21	72.87	74.27	75.24	73.97	74.03		86.93	85.49	86.53	89.15	87.52	89.01	87.66	89.11	88.16
20	85.52	86.53	86.34	85.03	85.67	86.10	84.01	84.50	83.56	86.54	84.60	86.59	84.22	84.30	87.62	88.01	85.89	85.49	76.64		98.29	87.40	94.13	90.22	94.72	94.67	95.16	94.59
21	84.88	86.55	85.71	84.17	85.26	85.25	83.58	84.96	83.13	85.69	83.72	86.18	84.24	82.96	85.94	86.34	84.59	85.08	76.91	97.43		85.89	92.24	88.81	92.85	92.78	93.26	92.68
22	75.28	75.82	75.60	75.85	75.82	76.76	81.32	79.02	79.68	75.57	75.09	78.67	78.22	76.02	75.10	77.58	76.59	75.31	73.45	77.33	75.85		89.08	86.35	88.99	87.54	88.51	88.13
23	78.14	81.27	78.93	79.16	81.51	79.85	84.98	83.26	83.22	80.34	79.90	84.29	84.32	79.81	80.56	81.22	80.99	79.62	76.30	82.60	81.48	82.79		90.46	95.43	94.20	94.58	94.74
24	75.23	77.14	75.23	75.22	77.14	79.08	78.62	77.21	79.05	76.70	74.48	79.81	79.37	78.11	75.24	77.66	78.30	76.70	72.71	79.05	77.88	75.09	77.06		91.75	90.45	90.29	91.62
25	81.60	81.34	80.01	80.48	81.34	81.80	89.17	90.01	90.38	80.93	80.72	90.16	89.36	81.76	80.50	81.39	82.73	80.51	77.52	83.36	82.70	78.94	83.24	78.16		97.75	97.69	99.43
26	81.11	80.61	80.46	80.92	80.13	80.84	88.54	88.98	88.92	80.90	80.22	89.94	89.35	81.32	79.76	80.66	82.30	79.77	77.50	83.16	82.27	79.88	83.89	78.62	93.79		96.57	97.69
27	80.96	81.16	80.06	81.23	80.69	80.92	88.79	90.04	90.03	80.98	80.77	90.58	89.42	79.50	80.31	81.21	80.98	81.21	78.49	84.08	83.65	77.28	82.19	77.48	95.66	92.30		97.05
28	81.75	81.95	81.33	80.86	81.95	82.17	88.82	88.03	89.46	81.99	80.87	88.17	87.18	80.04	78.98	80.84	81.03	79.24	78.10	82.13	81.70	78.99	83.06	77.04	93.25	90.36	91.95	

	Nucleotide similarity (%)																											

*Note:* (1): O-VN-CTU-VL01; (2): O-VN-CTU-VL02; (3): O-VN-CTU-TV01; (4): O-VN-CTU-TV02; (5): O-VN-CTU-TV03; (6): O-VN-CTU-TV04; (7): O-VN-CTU-BT01; (8): O-VN-CTU-BT02; (9): O-VN-CTU-BT03; (10): O-VN-CTU-BT04; (11): O-VN-CTU-BT05; (12): HQ116173.1_O/CAM/5/2006; (13): HQ116274.1_O/VIT/7/2004; (14): HQ116256.1_O/TAI/1/2009; (15): HQ116278-O/VIT/4/2005; (16): JQ070319.1_MYA/3/2010; (17): JQ070325.1_MOG/CO3/2010; (18): KC438373.1_SKR/12/2010; (19): AF154271.1_Tau-YuanTW97, (20): AJ294906.1_O/CAM/11/94; (21): AJ294929.1_O/VIT/2/97; (22): AJ303481.1_O_ALG/1/99; (23): AJ303511.1_O_KEN/83/79; (24): K01201.1_O1Campos/_Brazil/71; (25): KC506471.1_O/IND129/2010; (26): KC506543.1_O/IND62/2011; (27): KY399464.1_O/VIT/9595/2015; (28): MT936117.1_IC491/2018-Ind2001e.

## Data Availability

All data generated or analyzed during this study are included in this published article.
